# Tetra-μ-benzoato-bis­{[*trans*-1-(2-pyrid­yl)-2-(4-pyrid­yl)ethyl­ene]zinc(II)}

**DOI:** 10.1107/S1600536809045048

**Published:** 2009-11-04

**Authors:** Young Joo Song, Soo-Won Lee, Kyung Hwan Jang, Cheal Kim, Youngmee Kim

**Affiliations:** a Department of Fine Chemistry, and Eco-Product and Materials Education Center, Seoul National University of Technology, Seoul 139-743, Republic of Korea; b Forest Practice Research Center, Korea Forest Research Institute, Pocheon 487-821, Republic of Korea; c Korea Forest Research Institute 44-3, Suwon 441-350, Republic of Korea; d Deaprtment of Chemistry and Nano Science, Ewha Womans University, Seoul 120-750, Republic of Korea

## Abstract

The paddle-wheel-type centrosymmetric dinuclear title complex, [Zn_2_(C_7_H_5_O_2_)_4_(C_12_H_10_N_2_)_2_], contains four bridging benzoate groups and two terminal *trans*-1-(2-pyrid­yl)-2-(4-pyrid­yl)ethyl­ene (*L*) ligands. The inversion center is located between the two Zn^II^ atoms. The octa­hedral coordination around the Zn^II^ atom, with four O atoms in the equatorial plane, is completed by an N atom of the *L* mol­ecule [Zn—N = 2.0198 (15) Å] and by the second Zn^II^ atom [Zn⋯Zn = 2.971 (8) Å]. The Zn^II^ atom is 0.372 Å out of the plane of the four coordinating O atoms.

## Related literature

For structures containing [Zn_2_(O_2_CPh)_4_], see: Necefoglu *et al.* (2002 ▶); Zeleňák *et al.* (2004 ▶); Karmakar *et al.* (2006 ▶); Ohmura *et al.* (2005 ▶). For the structures of copper(II) and zinc(II) benzoates with quinoxaline, 6-methyl­quinoline, 3-methyl­quinoline, and di-2-pyridyl ketone, see: Lee *et al.* (2008 ▶); Yu *et al.* (2008 ▶, 2009 ▶); Park *et al.* (2008 ▶); Shin *et al.* (2009 ▶). For transition metal ions as the major cation contributors to the inorganic composition of natural water and biological fluids, see: Daniele *et al.* (2008 ▶); Parkin (2004 ▶); Tshuva & Lippard (2004 ▶). 
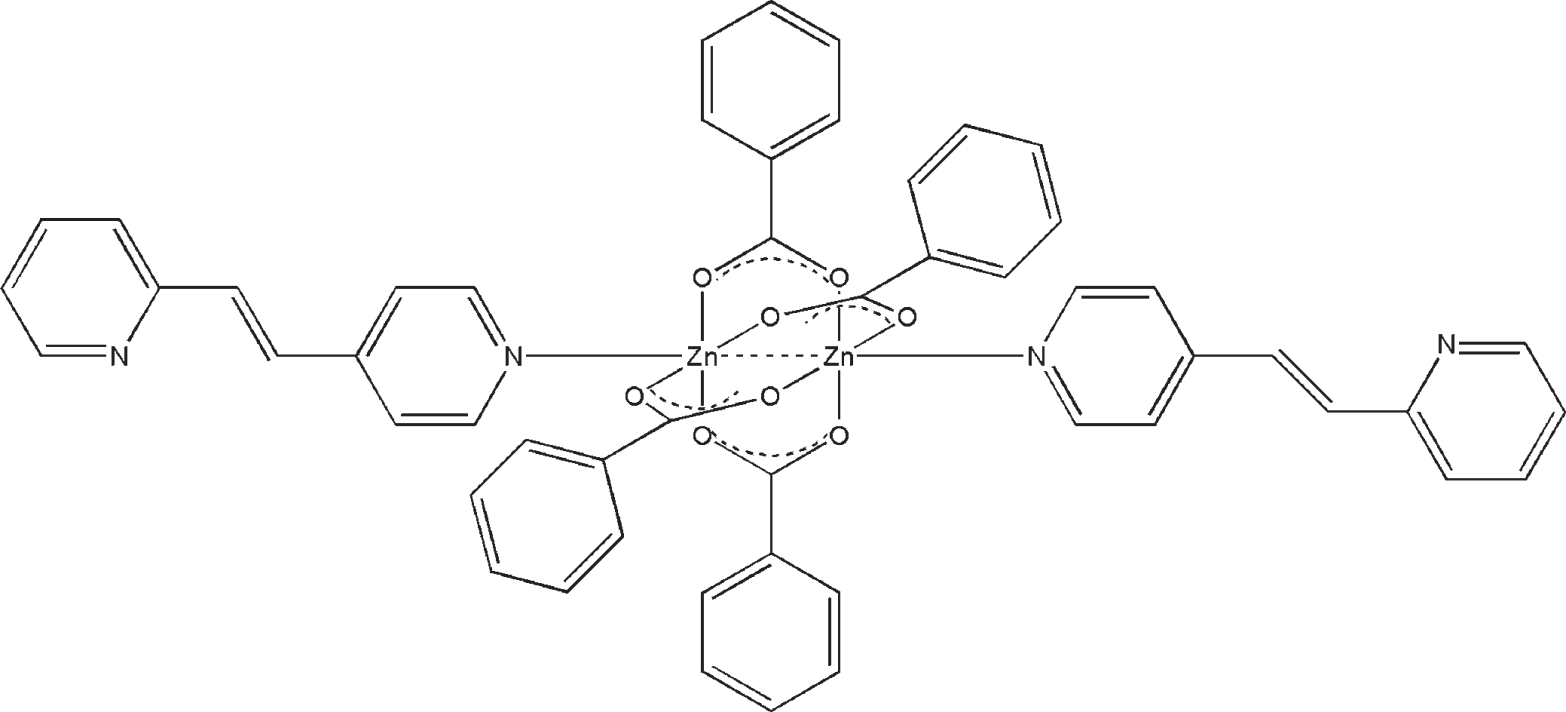


## Experimental

### 

#### Crystal data

[Zn_2_(C_7_H_5_O_2_)_4_(C_12_H_10_N_2_)_2_]
*M* *_r_* = 979.66
Monoclinic, 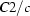

*a* = 24.919 (6) Å
*b* = 12.186 (3) Å
*c* = 15.742 (4) Å
β = 109.857 (4)°
*V* = 4496.0 (19) Å^3^
*Z* = 4
Mo *K*α radiation
μ = 1.13 mm^−1^
*T* = 293 K
0.20 × 0.15 × 0.15 mm

#### Data collection

Bruker SMART CCD diffractometer
Absorption correction: multi-scan (*SADABS*; Bruker, 1997 ▶) *T*_min_ = 0.816, *T*_max_ = 0.884
12326 measured reflections
4416 independent reflections
2947 reflections with *I* > 2σ(*I*)
*R*_int_ = 0.039

#### Refinement

*R*[*F* ^2^ > 2σ(*F* ^2^)] = 0.039
*wR*(*F* ^2^) = 0.090
*S* = 1.03
4416 reflections
298 parameters
H-atom parameters constrained
Δρ_max_ = 0.26 e Å^−3^
Δρ_min_ = −0.27 e Å^−3^

### 

Data collection: *SMART* (Bruker, 1997 ▶); cell refinement: *SAINT* (Bruker, 1997 ▶); data reduction: *SAINT*; program(s) used to solve structure: *SHELXS97* (Sheldrick, 2008 ▶); program(s) used to refine structure: *SHELXL97* (Sheldrick, 2008 ▶); molecular graphics: *SHELXTL* (Sheldrick, 2008 ▶); software used to prepare material for publication: *SHELXTL*.

## Supplementary Material

Crystal structure: contains datablocks I, global. DOI: 10.1107/S1600536809045048/dn2505sup1.cif

Structure factors: contains datablocks I. DOI: 10.1107/S1600536809045048/dn2505Isup2.hkl

Additional supplementary materials:  crystallographic information; 3D view; checkCIF report

## Figures and Tables

**Fig. 1.**
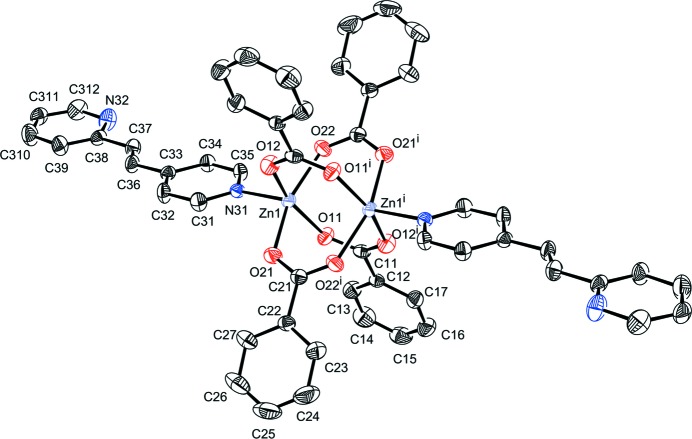
The structure of the title compound showing the atom-labeling scheme. Displacement ellipsoids are shown at the 30% probability level. H atoms have been omitted for clarity. [Symmetry code: (i) -x+1, -y+1, -z+1].

## supplementary crystallographic information

### Comment

A great attention has been paid to transition metal ions as the major cation contributors to the inorganic composition of natural water and biological fluids (Daniele, *et al.*, 2008; Parkin, 2004; Tshuva & Lippard, 2004). While the main attention was focused on the interaction of transition metal ions with biologically active molecules such as amino acids, proteins, sugars, nucleotides *etc*, the study on the interaction of the transition metal ions with fulvic acids and humic acids, mainly found in soil, is about to start. As models to examine the interaction, therefore, we have previously used copper(II) and zinc(II) benzoates as building blocks and reported the structures of copper(II) and zinc(II) benzoates with quinoxaline, 6-methylquinoline, 3-methylquinoline, and di-2-pyridyl ketone (Lee, *et al.*, 2008; Yu, *et al.*, 2008; Park, *et al.*, 2008; Shin, *et al.*, 2009; Yu, *et al.*, 2009). The related paddle-wheel type structures for Zn complexes have been previouly reported (Necefoglu *et al.*, 2002; Zeleňák, *et al.*, 2004; Karmakar, *et al.*, 2006; Ohmura, *et al.*, 2005). In this work, we have employed zinc(II) benzoate as a building block and *trans*-1-(2-pyridyl)-2-(4-pyridyl)ethylene as a ligand. We report hereon the structure of new zinc(II) benzoate with *trans*-1-(2-pyridyl)-2-(4-pyridyl)ethylene.

Asymmetric unit contains half of whole molecule, and there is an inversion center in the middle of Zn···Zn bond. Symmetric operation (1-*x*, 1-*y* , 1-*z*) produces a paddle-wheel type dinuclear zinc-benzoate complex (Fig. 1). The paddle-wheel type dinuclear complex is constructed by four bridging benzoate groups and two terminal *L* ligands (*L* = *trans*-1-(2-pyridyl)-2-(4-pyridyl)ethylene). The octahedral coordination around the zinc atom, with four O atoms in the equatorial plane, is completed by nitrogen atom of *L* molecule (Zn—N 2.0198 (15) Å) and by the second zinc atom (Zn···Zn 2.971 (8) Å). The zinc atom is 0.372 Å out of the plane of the four oxygen atoms.

### Experimental

30.4 mg (0.1 mmol) of Zn(NO_3_)_2_^.^6H_2_O and 28.0 mg (0.2 mmol) of C_6_H_5_COONH_4_ were dissolved in 4 ml H_2_O and carefully layered by 4 ml me thanol solution of *trans*-1-(2-pyridyl)-2-(4-pyridyl)ethylene (37.6 mg, 0.2 mmol). Suitable crystals of the title compound for X-ray analysis were obtained in a few weeks.

### Refinement

H atoms were placed in calculated positions with C—H distances of 0.93 Å. They were included in the refinement in a riding-motion approximation with Uĩso~(H) = 1.2U~eq~(C).

### Crystal data

**Table:**

[Zn_2_(C_7_H_5_O_2_)_4_(C_12_H_10_N_2_)_2_]	*F*(000) = 2016
*M**_r_* = 979.66	*D*_x_ = 1.447 Mg m^−^^3^
Monoclinic, *C*2/*c*	Mo *K*α radiation, λ = 0.71073 Å
Hall symbol: -C 2yc	Cell parameters from 1818 reflections
*a* = 24.919 (6) Å	θ = 2.5–19.6°
*b* = 12.186 (3) Å	µ = 1.13 mm^−^^1^
*c* = 15.742 (4) Å	*T* = 293 K
β = 109.857 (4)°	Block, colorless
*V* = 4496.0 (19) Å^3^	0.20 × 0.15 × 0.15 mm
*Z* = 4	

### Data collection

**Table:**

Bruker SMART CCD diffractometer	4416 independent reflections
Radiation source: fine-focus sealed tube	2947 reflections with *I* > 2σ(*I*)
graphite	*R*_int_ = 0.039
φ and ω scans	θ_max_ = 26.0°, θ_min_ = 1.9°
Absorption correction: multi-scan (*SADABS*; Bruker, 1997)	*h* = −20→30
*T*_min_ = 0.816, *T*_max_ = 0.884	*k* = −15→15
12326 measured reflections	*l* = −19→16

### Refinement

**Table:**

Refinement on *F*^2^	Primary atom site location: structure-invariant direct methods
Least-squares matrix: full	Secondary atom site location: difference Fourier map
*R*[*F*^2^ > 2σ(*F*^2^)] = 0.039	Hydrogen site location: inferred from neighbouring sites
*wR*(*F*^2^) = 0.090	H-atom parameters constrained
*S* = 1.03	*w* = 1/[σ^2^(*F*_o_^2^) + (0.0205*P*)^2^ + 1.48*P*] where *P* = (*F*_o_^2^ + 2*F*_c_^2^)/3
4416 reflections	(Δ/σ)_max_ = 0.001
298 parameters	Δρ_max_ = 0.26 e Å^−^^3^
0 restraints	Δρ_min_ = −0.26 e Å^−^^3^

### Special details

**Table:**

Geometry. All esds (except the esd in the dihedral angle between two l.s. planes) are estimated using the full covariance matrix. The cell esds are taken into account individually in the estimation of esds in distances, angles and torsion angles; correlations between esds in cell parameters are only used when they are defined by crystal symmetry. An approximate (isotropic) treatment of cell esds is used for estimating esds involving l.s. planes.
Refinement. Refinement of F^2^ against ALL reflections. The weighted R-factor wR and goodness of fit S are based on F^2^, conventional R-factors R are based on F, with F set to zero for negative F^2^. The threshold expression of F^2^ > 2sigma(F^2^) is used only for calculating R-factors(gt) etc. and is not relevant to the choice of reflections for refinement. R-factors based on F^2^ are statistically about twice as large as those based on F, and R- factors based on ALL data will be even larger.

### Fractional atomic coordinates and isotropic or equivalent isotropic displacement parameters (Å^2^)

**Table:**

	*x*	*y*	*z*	*U*_iso_*/*U*_eq_	
Zn1	0.545232 (12)	0.50480 (2)	0.590326 (19)	0.03852 (11)	
O11	0.48081 (8)	0.42174 (16)	0.61458 (13)	0.0536 (5)	
O12	0.58816 (8)	0.58560 (17)	0.51974 (13)	0.0589 (5)	
O21	0.56818 (8)	0.35925 (15)	0.54865 (13)	0.0549 (5)	
O22	0.50102 (8)	0.64874 (15)	0.58505 (13)	0.0581 (6)	
N31	0.60407 (9)	0.52228 (16)	0.71558 (14)	0.0391 (5)	
N32	0.75201 (11)	0.7049 (2)	1.18938 (17)	0.0710 (8)	
C11	0.43281 (12)	0.3906 (2)	0.56234 (19)	0.0420 (7)	
C12	0.39861 (11)	0.3173 (2)	0.60095 (18)	0.0399 (6)	
C13	0.41930 (13)	0.2875 (3)	0.6908 (2)	0.0584 (8)	
H13	0.4539	0.3159	0.7282	0.070*	
C14	0.38945 (18)	0.2165 (3)	0.7258 (3)	0.0805 (11)	
H14	0.4041	0.1973	0.7865	0.097*	
C15	0.33831 (18)	0.1736 (3)	0.6724 (3)	0.0803 (11)	
H15	0.3184	0.1251	0.6963	0.096*	
C16	0.31681 (14)	0.2032 (3)	0.5828 (3)	0.0747 (10)	
H16	0.2821	0.1745	0.5459	0.090*	
C17	0.34645 (12)	0.2755 (2)	0.5472 (2)	0.0563 (8)	
H17	0.3312	0.2962	0.4869	0.068*	
C21	0.53915 (12)	0.3100 (2)	0.47807 (19)	0.0423 (6)	
C22	0.55306 (11)	0.1917 (2)	0.46933 (19)	0.0450 (7)	
C23	0.51906 (15)	0.1303 (3)	0.3980 (3)	0.0773 (11)	
H23	0.4894	0.1636	0.3525	0.093*	
C24	0.5289 (2)	0.0196 (3)	0.3940 (4)	0.1091 (17)	
H24	0.5051	−0.0218	0.3464	0.131*	
C25	0.5726 (2)	−0.0295 (3)	0.4583 (4)	0.1087 (17)	
H25	0.5786	−0.1044	0.4551	0.130*	
C26	0.6079 (2)	0.0306 (3)	0.5279 (3)	0.0902 (13)	
H26	0.6384	−0.0034	0.5714	0.108*	
C27	0.59863 (14)	0.1418 (3)	0.5344 (2)	0.0628 (9)	
H27	0.6228	0.1826	0.5820	0.075*	
C31	0.65674 (12)	0.4837 (2)	0.73577 (19)	0.0531 (8)	
H31	0.6658	0.4437	0.6922	0.064*	
C32	0.69861 (12)	0.5004 (2)	0.81848 (19)	0.0568 (8)	
H32	0.7351	0.4728	0.8292	0.068*	
C33	0.68646 (11)	0.5579 (2)	0.88547 (17)	0.0412 (7)	
C34	0.63111 (11)	0.5948 (2)	0.86490 (17)	0.0473 (7)	
H34	0.6203	0.6323	0.9080	0.057*	
C35	0.59211 (11)	0.5760 (2)	0.78060 (17)	0.0456 (7)	
H35	0.5552	0.6024	0.7681	0.055*	
C36	0.73130 (12)	0.5774 (2)	0.97322 (18)	0.0505 (7)	
H36	0.7680	0.5540	0.9794	0.061*	
C37	0.72419 (12)	0.6248 (2)	1.04352 (18)	0.0509 (8)	
H37	0.6872	0.6455	1.0378	0.061*	
C38	0.76886 (13)	0.6485 (2)	1.13035 (18)	0.0473 (7)	
C39	0.82430 (14)	0.6161 (3)	1.1499 (2)	0.0647 (9)	
H39	0.8352	0.5770	1.1077	0.078*	
C310	0.86366 (15)	0.6418 (3)	1.2323 (2)	0.0819 (12)	
H310	0.9014	0.6194	1.2467	0.098*	
C311	0.84732 (15)	0.7006 (3)	1.2933 (2)	0.0639 (9)	
H311	0.8735	0.7201	1.3491	0.077*	
C312	0.79166 (16)	0.7295 (3)	1.2699 (2)	0.0722 (10)	
H312	0.7802	0.7684	1.3116	0.087*	

### Atomic displacement parameters (Å^2^)

**Table:**

	*U*^11^	*U*^22^	*U*^33^	*U*^12^	*U*^13^	*U*^23^
Zn1	0.03822 (19)	0.04063 (17)	0.03006 (17)	−0.00220 (15)	0.00295 (12)	−0.00148 (14)
O11	0.0470 (12)	0.0593 (12)	0.0528 (12)	−0.0127 (10)	0.0146 (10)	−0.0025 (10)
O12	0.0622 (13)	0.0699 (13)	0.0446 (12)	−0.0098 (11)	0.0180 (10)	0.0091 (11)
O21	0.0599 (13)	0.0486 (11)	0.0522 (13)	0.0073 (10)	0.0140 (10)	−0.0082 (10)
O22	0.0580 (13)	0.0474 (11)	0.0584 (14)	0.0102 (10)	0.0063 (11)	0.0015 (10)
N31	0.0405 (13)	0.0400 (12)	0.0331 (12)	−0.0012 (10)	0.0078 (10)	−0.0030 (9)
N32	0.0614 (18)	0.105 (2)	0.0411 (15)	−0.0057 (16)	0.0108 (13)	−0.0143 (15)
C11	0.0491 (18)	0.0344 (14)	0.0460 (17)	0.0026 (13)	0.0207 (14)	−0.0019 (13)
C12	0.0426 (16)	0.0373 (14)	0.0431 (16)	0.0024 (12)	0.0190 (13)	−0.0015 (12)
C13	0.062 (2)	0.0643 (19)	0.052 (2)	−0.0050 (17)	0.0227 (16)	0.0023 (16)
C14	0.100 (3)	0.086 (3)	0.066 (2)	0.002 (2)	0.042 (2)	0.022 (2)
C15	0.094 (3)	0.057 (2)	0.111 (3)	−0.002 (2)	0.062 (3)	0.015 (2)
C16	0.056 (2)	0.069 (2)	0.102 (3)	−0.0149 (18)	0.031 (2)	−0.007 (2)
C17	0.0473 (19)	0.0579 (18)	0.062 (2)	−0.0040 (15)	0.0168 (16)	−0.0014 (16)
C21	0.0444 (17)	0.0416 (14)	0.0452 (17)	0.0015 (13)	0.0206 (14)	0.0010 (13)
C22	0.0479 (17)	0.0401 (14)	0.0545 (18)	0.0015 (13)	0.0270 (14)	−0.0016 (13)
C23	0.068 (2)	0.059 (2)	0.097 (3)	−0.0027 (18)	0.016 (2)	−0.0217 (19)
C24	0.103 (4)	0.063 (3)	0.163 (5)	−0.014 (2)	0.046 (3)	−0.050 (3)
C25	0.123 (4)	0.042 (2)	0.192 (6)	0.007 (2)	0.094 (4)	−0.003 (3)
C26	0.103 (3)	0.064 (2)	0.121 (4)	0.035 (2)	0.061 (3)	0.034 (2)
C27	0.071 (2)	0.061 (2)	0.062 (2)	0.0168 (17)	0.0303 (18)	0.0140 (16)
C31	0.0490 (18)	0.0643 (19)	0.0413 (16)	0.0072 (15)	0.0093 (13)	−0.0153 (14)
C32	0.0391 (16)	0.074 (2)	0.0488 (18)	0.0100 (16)	0.0043 (13)	−0.0124 (17)
C33	0.0440 (17)	0.0416 (15)	0.0331 (15)	−0.0033 (13)	0.0067 (12)	−0.0023 (12)
C34	0.0447 (17)	0.0595 (17)	0.0356 (15)	0.0031 (14)	0.0110 (13)	−0.0087 (13)
C35	0.0360 (16)	0.0582 (17)	0.0374 (16)	0.0044 (14)	0.0056 (12)	−0.0004 (14)
C36	0.0397 (17)	0.0608 (18)	0.0410 (17)	0.0010 (14)	0.0009 (13)	−0.0077 (14)
C37	0.0445 (18)	0.0638 (19)	0.0371 (16)	−0.0022 (14)	0.0045 (13)	−0.0036 (14)
C38	0.0547 (19)	0.0500 (16)	0.0324 (16)	−0.0109 (14)	0.0083 (14)	−0.0012 (13)
C39	0.059 (2)	0.077 (2)	0.0444 (18)	0.0088 (17)	0.0002 (16)	−0.0159 (16)
C310	0.062 (2)	0.099 (3)	0.062 (2)	0.005 (2)	−0.0074 (19)	−0.013 (2)
C311	0.071 (2)	0.069 (2)	0.0367 (18)	−0.0147 (19)	−0.0020 (16)	−0.0016 (16)
C312	0.079 (3)	0.096 (3)	0.0382 (18)	−0.009 (2)	0.0162 (17)	−0.0134 (18)

### Geometric parameters (Å, °)

**Table:**

Zn1—N31	2.029 (2)	C23—C24	1.376 (5)
Zn1—O12	2.039 (2)	C23—H23	0.9300
Zn1—O21	2.0392 (19)	C24—C25	1.349 (6)
Zn1—O11	2.0407 (19)	C24—H24	0.9300
Zn1—O22	2.0580 (19)	C25—C26	1.362 (6)
Zn1—Zn1^i^	2.9711 (8)	C25—H25	0.9300
O11—C11	1.258 (3)	C26—C27	1.385 (4)
O12—C11^i^	1.252 (3)	C26—H26	0.9300
O21—C21	1.254 (3)	C27—H27	0.9300
O22—C21^i^	1.251 (3)	C31—C32	1.379 (4)
N31—C31	1.327 (3)	C31—H31	0.9300
N31—C35	1.331 (3)	C32—C33	1.383 (4)
N32—C38	1.333 (4)	C32—H32	0.9300
N32—C312	1.350 (4)	C33—C34	1.381 (3)
C11—O12^i^	1.252 (3)	C33—C36	1.471 (3)
C11—C12	1.498 (4)	C34—C35	1.372 (3)
C12—C13	1.380 (4)	C34—H34	0.9300
C12—C17	1.385 (4)	C35—H35	0.9300
C13—C14	1.372 (4)	C36—C37	1.313 (4)
C13—H13	0.9300	C36—H36	0.9300
C14—C15	1.370 (5)	C37—C38	1.468 (3)
C14—H14	0.9300	C37—H37	0.9300
C15—C16	1.375 (5)	C38—C39	1.368 (4)
C15—H15	0.9300	C39—C310	1.371 (4)
C16—C17	1.385 (4)	C39—H39	0.9300
C16—H16	0.9300	C310—C311	1.366 (5)
C17—H17	0.9300	C310—H310	0.9300
C21—O22^i^	1.251 (3)	C311—C312	1.355 (4)
C21—C22	1.500 (4)	C311—H311	0.9300
C22—C23	1.375 (4)	C312—H312	0.9300
C22—C27	1.385 (4)		
			
N31—Zn1—O12	98.00 (8)	C24—C23—H23	120.0
N31—Zn1—O21	102.41 (8)	C22—C23—H23	120.0
O12—Zn1—O21	89.34 (8)	C25—C24—C23	120.7 (4)
N31—Zn1—O11	103.00 (8)	C25—C24—H24	119.7
O12—Zn1—O11	158.97 (8)	C23—C24—H24	119.7
O21—Zn1—O11	87.31 (8)	C24—C25—C26	120.1 (4)
N31—Zn1—O22	98.62 (8)	C24—C25—H25	119.9
O12—Zn1—O22	86.52 (9)	C26—C25—H25	119.9
O21—Zn1—O22	158.93 (8)	C25—C26—C27	120.4 (4)
O11—Zn1—O22	89.19 (8)	C25—C26—H26	119.8
N31—Zn1—Zn1^i^	175.50 (6)	C27—C26—H26	119.8
O12—Zn1—Zn1^i^	82.26 (6)	C26—C27—C22	119.4 (3)
O21—Zn1—Zn1^i^	82.08 (6)	C26—C27—H27	120.3
O11—Zn1—Zn1^i^	76.71 (6)	C22—C27—H27	120.3
O22—Zn1—Zn1^i^	76.89 (5)	N31—C31—C32	122.9 (3)
C11—O11—Zn1	131.38 (19)	N31—C31—H31	118.5
C11^i^—O12—Zn1	124.19 (18)	C32—C31—H31	118.5
C21—O21—Zn1	124.11 (17)	C31—C32—C33	120.2 (3)
C21^i^—O22—Zn1	130.32 (18)	C31—C32—H32	119.9
C31—N31—C35	116.8 (2)	C33—C32—H32	119.9
C31—N31—Zn1	121.66 (18)	C34—C33—C32	116.5 (2)
C35—N31—Zn1	121.47 (18)	C34—C33—C36	123.2 (2)
C38—N32—C312	117.7 (3)	C32—C33—C36	120.3 (3)
O12^i^—C11—O11	125.1 (3)	C35—C34—C33	119.7 (3)
O12^i^—C11—C12	117.5 (2)	C35—C34—H34	120.1
O11—C11—C12	117.4 (3)	C33—C34—H34	120.1
C13—C12—C17	118.4 (3)	N31—C35—C34	123.8 (3)
C13—C12—C11	120.5 (2)	N31—C35—H35	118.1
C17—C12—C11	121.1 (3)	C34—C35—H35	118.1
C14—C13—C12	120.8 (3)	C37—C36—C33	125.9 (3)
C14—C13—H13	119.6	C37—C36—H36	117.1
C12—C13—H13	119.6	C33—C36—H36	117.1
C15—C14—C13	120.8 (3)	C36—C37—C38	126.5 (3)
C15—C14—H14	119.6	C36—C37—H37	116.8
C13—C14—H14	119.6	C38—C37—H37	116.8
C14—C15—C16	119.1 (3)	N32—C38—C39	121.7 (3)
C14—C15—H15	120.4	N32—C38—C37	115.6 (3)
C16—C15—H15	120.4	C39—C38—C37	122.8 (3)
C15—C16—C17	120.4 (3)	C38—C39—C310	119.3 (3)
C15—C16—H16	119.8	C38—C39—H39	120.3
C17—C16—H16	119.8	C310—C39—H39	120.3
C16—C17—C12	120.4 (3)	C311—C310—C39	119.8 (3)
C16—C17—H17	119.8	C311—C310—H310	120.1
C12—C17—H17	119.8	C39—C310—H310	120.1
O22^i^—C21—O21	125.2 (2)	C312—C311—C310	117.8 (3)
O22^i^—C21—C22	117.4 (2)	C312—C311—H311	121.1
O21—C21—C22	117.3 (2)	C310—C311—H311	121.1
C23—C22—C27	119.2 (3)	N32—C312—C311	123.6 (3)
C23—C22—C21	120.0 (3)	N32—C312—H312	118.2
C27—C22—C21	120.8 (3)	C311—C312—H312	118.2
C24—C23—C22	120.1 (4)		
			
O12^i^—C11—C12—C13	179.8 (3)	C21—C22—C27—C26	175.3 (3)
O11—C11—C12—C13	1.0 (4)	C35—N31—C31—C32	−2.1 (4)
O12^i^—C11—C12—C17	1.5 (4)	N31—C31—C32—C33	1.1 (5)
O11—C11—C12—C17	−177.2 (3)	C31—C32—C33—C34	0.9 (4)
C17—C12—C13—C14	1.3 (5)	C31—C32—C33—C36	−178.9 (3)
C11—C12—C13—C14	−177.0 (3)	C32—C33—C34—C35	−1.7 (4)
C12—C13—C14—C15	−0.1 (5)	C36—C33—C34—C35	178.1 (3)
C13—C14—C15—C16	−0.5 (6)	C31—N31—C35—C34	1.3 (4)
C14—C15—C16—C17	−0.1 (6)	C33—C34—C35—N31	0.7 (4)
C15—C16—C17—C12	1.2 (5)	C34—C33—C36—C37	4.5 (5)
C13—C12—C17—C16	−1.8 (4)	C32—C33—C36—C37	−175.6 (3)
C11—C12—C17—C16	176.5 (3)	C33—C36—C37—C38	−177.6 (3)
O22^i^—C21—C22—C23	−5.7 (4)	C312—N32—C38—C39	0.0 (5)
O21—C21—C22—C23	173.3 (3)	C312—N32—C38—C37	−179.4 (3)
O22^i^—C21—C22—C27	176.9 (3)	C36—C37—C38—N32	175.1 (3)
O21—C21—C22—C27	−4.1 (4)	C36—C37—C38—C39	−4.4 (5)
C27—C22—C23—C24	2.9 (6)	N32—C38—C39—C310	0.2 (5)
C21—C22—C23—C24	−174.5 (4)	C37—C38—C39—C310	179.6 (3)
C22—C23—C24—C25	−1.6 (7)	C38—C39—C310—C311	−0.9 (5)
C23—C24—C25—C26	−0.5 (8)	C39—C310—C311—C312	1.3 (5)
C24—C25—C26—C27	1.4 (7)	C38—N32—C312—C311	0.4 (5)
C25—C26—C27—C22	0.0 (6)	C310—C311—C312—N32	−1.1 (5)
C23—C22—C27—C26	−2.1 (5)		

Symmetry codes: (i) −*x*+1, −*y*+1, −*z*+1.
